# Food Allergy: Epidemiology, Pathogenesis, Diagnosis, and Treatment

**DOI:** 10.3390/jcm13196010

**Published:** 2024-10-09

**Authors:** Angela Rizzi, Sebastiano Gangemi

**Affiliations:** 1 UOSD Allergologia e Immunologia Clinica, Dipartimento di Scienze Mediche e Chirurgiche, Fondazione Policlinico Universitario A. Gemelli IRCCS, 00168 Rome, Italy; 2 Department of Clinical and Experimental Medicine, School and Operative Unit of Allergy and Clinical Immunology, University of Messina, 98125 Messina, Italy; gangemis@unime.it

Food allergy (FA) is a complex disease characterized by an immunological adverse reaction that may occur following exposure to apparently harmless food allergens. Generally, the immune response can be classified into three types: immunoglobulin E-mediated, non-immunoglobulin E-mediated, or with a mixed pathophysiologic mechanism, including eosinophilic esophagitis [1].

FA is one of the most frequent chronic diseases affecting both children and adults, with a considerable negative impact on the quality of life, especially in childhood, becoming an important global health problem [2].

However, despite a constant worldwide increase in specialistic visits and hospital admissions for anaphylaxis, urticaria, and angioedema related to FA, there are still few epidemiological studies in developing countries and geographical areas with a high variety of culinary habits and customs [3].

Research in recent decades focused on identifying strategies to contain the food allergy epidemic and provided a better understanding of the underlying pathogenetic mechanisms with potential therapeutic implications.

This Special Issue collected five papers (summarized in Table 1) and focused on prevalence (contribution 1), pediatric population (contribution 2), pathophysiological mechanisms (contribution 3), impact on quality of life (contribution 4), and nutritional therapeutic strategies (contribution 5).

The original article by Arámburo-Gálvez et al. (contribution 1) described the prevalence of FA through a cross-sectional survey to which 810 parents of Mexican preschoolers (aged 3–6 years old) responded. Three hundred and twenty-nine questionaries (40.61%) reported a personal history of allergy, while at least two allergic diseases were described in one hundred and forty-one responders (17.4%). The two most frequent atopic manifestations were allergic rhinitis (17.65%) and atopic dermatitis (14.32%). The survey revealed that 1.6% (*n* = 13) of enrolled responders had immediate-type FA. Nine of the thirteen allergic children presented symptoms and signs compatible with food-induced anaphylaxis (1.11%). This estimate is within the range of most prevalence rates based on global surveys (from 0.4% to 17.6%), lower than that reported in another study conducted in western Mexico (3.5%), probably due to the high geographic variability of this area [4]. The foods most frequently involved in the induction of food allergic reactions were milk (0.49%), strawberry (0.37%), eggs, soy, and chocolate (0.25%). More than half of patients (61.53%) had a skin reaction (urticaria, redness on the skin, angioedema), followed by gastrointestinal symptoms (vomit, abdominal pain, and diarrhea). Children with “immediate-type FA” had more atopic backgrounds and a positive parental history of allergies. Furthermore, the authors confirmed the low rate of adrenaline prescriptions despite immediate FA requiring access to emergency departments, as reported by Bedolla-Barajas et al. [4] and Jares et al. [5].

Latex allergy continues to be a major global health problem, mainly due to exposure to the allergen in at-risk individuals (such as healthcare workers) and patients (history of repeated surgeries, patients with urogenital malformations, and spina bifida) [6,7].

Arasi et al. (contribution 2) performed a comprehensive review of the current knowledge on epidemiology, diagnostics, and management in terms of prevention and therapy for pediatric latex allergy.

The section of the manuscript focused on the need to implement preventive measures at all levels deserves emphasis. Both the general population and at-risk subjects are the target of primary prevention strategies. Secondary prevention measures focus on the key role of the general practitioner/pediatrician in the diagnostic process. Finally, the most important goal of tertiary prevention is to avoid severe allergic reactions, in particular, anaphylactic reactions, by providing the patient with a management plan for allergic reactions that includes, if necessary, the prescription of an adrenaline autoinjector. The adoption of comprehensive educational and preventive measures in at-risk environments (such as hospitals) is crucial to minimize/eliminate the risk of accidental exposure to the allergen.

A deeper understanding of the complex pathogenic mechanisms underlying food allergy is essential for the development of more effective and personalized therapeutic strategies.

The narrative review focused on the emerging role of alarmins in food allergy, reported in this Special Issue (contribution 3), and analytically described the pathomechanisms of alarmins in FA. Cytoplasmic and granule-derived alarmins could become diagnostic and therapeutic biomarkers (such as response to an exclusion diet). Interleukin 33, interleukin 25, and thymic stromal lymphopoietin could represent a therapeutic target. Many studies on the role of alarmins in FA have been conducted in animal models and have never applied to humans. Further investigations, tests in human models, and studies on larger cohorts could help develop new diagnostic and therapeutic strategies for better management of this disease.

FA can negatively impact the patient’s quality of life (QoL) and health-related quality of life (HRQoL) [8].

Pasioti et al. (contribution 4) assessed QoL of children aged 8–12 years old, teenagers, and adults with a presumed tree nut and/or peanut allergy through a dedicated questionnaire. A total of 106 respondents (46 children, 26 adolescents, and 34 adults) were included in the analysis. The survey revealed that tree nut and/or peanut allergy moderately affected QoL of patients with FA, with adults reporting the best outcomes and teenagers the worst. Fear of accidental reactions and frustration with allergies characterized the emotional impact of children. Constant vigilance regarding safe food choices and social restrictions mainly influenced the QoL in adolescents and adults.

After diagnosing a food allergy (FA), an elimination diet in regard to the allergen involved is one of the central pillars in the management of these patients to prevent even severe clinical manifestations [9].

Pitsios et al. (contribution 5) systematically reviewed the evidence on the efficacy of allergy-based elimination diets in patients with eosinophilic esophagitis (EoE). The authors selected sixteen studies fulfilling the quality assessment criteria and exploring the relationship among skin-prick tests, atopy patch tests, serum-specific IgE, and IgG4 with recognized triggers of EoE. The selected studies allowed the authors to highlight an efficacy of 66–88.3% of elimination diets guided by allergy testing (combining the results of IgE detection with APT); a fact that would make the treatment of EoE with diets guided by allergy testing not superior to empirical diets.

In summary, this Special Issue provides a valuable collection that gives insight into epidemiology, pathogenesis, diagnosis, and treatment of food allergy.

## Figures and Tables

**Table 1:** Summary of the papers published in the Special Issue titled “Food Allergy: Epidemiology, Pathogenesis, Diagnosis, and Treatment”.

Contribution Number	Reference	Type of Publication	Number of Authors	Number of Affiliations	Locations of Authors’ Affiliation	Topic of Allergy
1	Arámburo-Gálvez, J.G. et al. Prevalence of Parent-Reported Food Allergy in a Mexican Pre-School Population. J Clin Med. 2023 Aug 3;12(15):5095.	Article	11	6	Mexico	Epidemiology
2	Arasi, S. et al. Latex Allergy in Children. J Clin Med. 2023 Dec 25;13(1):124.	Review	14	11	Italy	Epidemiology, Pathogenesis, Diagnosis, and Treatment
3	Rizzi, A. et al. Emerging Role of Alarmins in Food Allergy: An Update on Pathophysiological Insights, Potential Use as Disease Biomarkers, and Therapeutic Implications. J Clin Med. 2023 Apr 4;12(7):2699.	Review	8	6	Italy	Pathogenesis, Diagnosis, and Treatment
4	Pasioti, M. et al. Impact of Presumed Tree Nut and Peanut Allergy on Quality of Life at Different Ages. J Clin Med. 2023 May 15;12(10):3472.	Article	7	5	Greece UK	Epidemiology, Quality of Life
5	Pitsios, C. et al. Allergy-Test-Based Elimination Diets for the Treatment of Eosinophilic Esophagitis: A Systematic Review of Their Efficacy. J Clin Med. 2022 Sep 24;11(19):5631.	Systematic Review	10	7	Cyprus Greece The Netherlands USA Italy	Treatment

## References

[1] Sampath V., Abrams E.M., Adlou B., Akdis C., Akdis M., Brough H.A., Chan S., Chatchatee P., Chinthrajah R.S., Cocco R.R. (2021). Food Allergy across the Globe. J. Allergy Clin. Immunol..

[2] Warren C.M., Jiang J., Gupta R.S. (2020). Epidemiology and Burden of Food Allergy. Curr. Allergy Asthma Rep..

[3] Jimenez-Garcia R., Lopez-de-Andres A., Hernandez-Barrera V., Zamorano-Leon J.J., Cuadrado-Corrales N., de Miguel-Diez J., Del-Barrio J.L., Jimenez-Sierra A., Carabantes-Alarcon D. (2024). Hospitalizations for Food-Induced Anaphylaxis between 2016 and 2021: Population-Based Epidemiologic Study. JMIR Public Health Surveill.

[4] Bedolla-Barajas M., Morales-Romero J., Sánchez-Magallón R., Valdez-Soto J.A., Bedolla-Pulido T.R., Meza-López C. (2023). Food Allergy among Mexican Infants and Preschoolers: Prevalence and Associated Factors. World J. Pediatr..

[5] Jares E.J., Cardona V., Gómez R.M., Bernstein J.A., Rosario Filho N.A., Cherrez-Ojeda I., Ensina L.F., De Falco A., Díaz M.C., Chávez Vereau P.A. (2023). Latin American Anaphylaxis Registry. World Allergy Organ. J..

[6] Wu M., McIntosh J., Liu J. (2016). Current Prevalence Rate of Latex Allergy: Why It Remains a Problem?. J. Occup. Health.

[7] Nucera E., Aruanno A., Rizzi A., Centrone M. (2020). Latex Allergy: Current Status and Future Perspectives. J. Asthma Allergy.

[8] Aaneland H., Larsen M.H., Helseth S., Wahl A.K. (2024). Quality Appraisal of Quality of Life Research in Children and Adolescents with Food Allergy: A Systematic Review. Int. Arch. Allergy Immunol..

[9] Rossi C.M., Lenti M.V., Merli S., Cena H., Di Sabatino A. (2023). Dietary Strategies in Adult Patients with Eosinophilic Esophagitis: A State-of-the-Art Review. Nutrients.

